# Estimation of genetic parameters and genetic trends for milk yield traits in Jamunapari goats in semiarid tropics

**DOI:** 10.1016/j.smallrumres.2017.05.004

**Published:** 2017-08

**Authors:** P.K. Rout, O. Matika, R. Kaushik, M.S. Dige, G. Dass, S.K. Singh

**Affiliations:** a Genetics and Breeding Division, ICAR- Central Institute for Research on Goats, Makhdoom, Farah, Mathura, 281122, Uttar Pradesh, India; b Genetics and Genomics Division, The Roslin Institute and R (D) SVS, University of Edinburgh, UK

**Keywords:** Goat, Animal model, Selective breeding, Milk production traits

## Abstract

The Jamunapari goats are reared as dairy animals in semi-arid conditions, therefore it is necessary to evaluate the genetic potential for the genetic improvement of milk production traits. The data comprised of 2217 phenotypic records for milk yield at 90 days (MY90) and 140 days (MY140), total milk yield (TMY) and lactation length (LL) obtained from the progeny of 173 sires and 446 dams during the period 1990–2013. The data were analysed using mixed linear models exploring random effects due to direct additive, maternal and permanent environment variance due to animal. The most appropriate genetic models for milk yield traits were those that included permanent environment effects due to the animal. The direct additive heritability estimates were 0.15 ± 0.05, 0.26 ± 0.07, 0.25 ± 0.08 for MY90, MY140 and TMY, respectively. The additive heritability estimate for LL was low and non-significant at 0.02 ± 0.03. The repeatability estimates were moderate to high ranging from 0.68 to 0.73 for milk yield traits. The repeatability for lactation length was 0.20 ± 0.03. Maternal variances were low ranging from 0.03 for MY90 to 0.13 for TMY. There was an increase in mean milk yield of 0.25, 0.70 and 0.72 kg/year respectively at 90 and 140 days, and for TMY. Genetic trends and phenotypic trends for MY90, MY140 and TMY were positive and indicated significant improvement in milk traits due to selective breeding.

## Highlights

• The heritability for milk yield traits were moderate to high.
• The repeatability estimates were moderate to high for milk yield traits.
• The genetic trends for milk yield traits were positive.
• Phenotypic trends for MY90, MY140 and TMY were positive.

## 1 Introduction

Genetic improvement programmes have focused on increasing various economically important traits for higher productivity and better income. Goat milk has potential human health benefit and plays significant role in nutraceutical, Ayurveda medicine formulation and fashion industry. Goat milk is attracting more attention due to health promoting properties and is used as human infant milk replacement food with no known allergies (Haenlein, 2004). In many household, goat milk is consumed and is a protein supplement for the most vulnerable groups such as women and children (Haenlein, 2004, Devendra, 2012). The economic and social importance of dairy goats in India is because of their ability to use poor vegetation in varied agro climatic regions and produce about 26% of world’s goat milk (Devendra, 2012). Therefore, goats are thus favoured as diary animal in the low-input systems and marginal environments due to special characteristics such as low capital investment, production costs and optimum use of meagre food resources (Devendra and Liang 2012). The Jamunapari goat is well known as one of the milk-producing Indian dairy breed in the subcontinent. These goats are tall, white in colour and large sized breed and adapted to semi-arid climatic condition (Rout et al., 2000). The Jamunapari goat has been used to upgrade milk production in other Asian breeds and elsewhere around the world (Rout et al., 2004).

Breed and genetic characterization for economically important traits for these goats are necessary in selection programmes to bring desired genetic improvement. The genetic parameters estimates for both milk and growth form essential inputs for future realistic breeding programmes. The knowledge of the (co)variance components and heritability estimates for milk production traits will support breeding strategies by selecting animals with superior genetic merit to optimize the response to selection and to improve dairy traits as desired (Sullivan et al., 1986, Barillet, 2007).

Genetic parameter estimation on milk yield traits has been carried out in different goat breeds (Belichon et al., 1999, Serradilla, 2001, Montaldo et al., 2010a, Wiggans et al., 1988, Wiggans, 1989, Majid et al., 1994). Moreover, the genetic parameter estimates for dairy goats are available for Mediterranean and Latin American countries (Barillet, 2007, Torres-Vazquez et al., 2010, Montaldo et al., 2010b). Furthermore, genetic parameters for yield traits in dairy goats have been estimated for other populations in South Africa, New Zealand and Norway (Muller et al., 2002, Morris et al., 2006, Andonov et al., 2007). The genetic parameters of milk yield traits have been reported in different local goats in different countries (Selvaggi and Dario, 2015, Mavrogenis et al., 1989, Brito et al., 2011, Kala and Prakash, 1990, Rabasco et al., 1993, Valencia et al., 2007). Despite the importance of goat milk for nutritional and livelihood security, there is still limited information on genetic and phenotypic parameters of milk yield and composition traits in different Indian indigenous breeds. Genetic trends for milk yield traits have been reported in French and American goat breeds (Clement et al., 2002, Wiggans et al., 2003). However, such information on genetic trends of milk yield traits for long-term selection experiment in Indian goat breeds has not been documented. We hope that the quantification of response to selection will facilitate a review of the selection objectives and management goals for our breeding programme. Therefore, the objectives of the present study were to determine the most appropriate models and estimate genetic parameters and genetic trends for milk yield traits for Jamunapari goats in semi-arid tropics.

## 2 Materials and methods

### 2.1 Herd description

Milk data and pedigree were available from 1990 to 2013 on Jamunapari goats maintained at the ICAR-Central Institute for Research on Goats (CIRG), Makhdoom, Mathura. The Jamunapari goats were introduced from their natural habitat, the Chakarnagar area of Etawah district of Uttar Pradesh, which is 150 km distant from CIRG, Mathura. The Jamunapari goat is a milk-producing breed with average body weight of 28.0 kg at 12 months of age and 1.46 kidding rate (Rout et al., 2000).

The goats were maintained under a semi-intensive system of management with 6–7 h of grazing and stall feeding with seasonally available green fodder *ad libitum*, supplemented with concentrate mixtures depending upon the status and age category of the animals. Generally, animals were housed separately according to their ages, sex, physiological status and health status. Controlled breeding was practiced with the does being bred during months of May to June and October to November followed by kidding in the months of October to November and March to April, respectively. Does were mated with bucks by natural mating twice at each oestrus. At kidding, each kid was assigned an identification number by ear tattooing and records of date of birth, sex, birth type and live body weights were taken. Kids were stall-fed up to weaning at 3 months of age, and then allowed to graze nearby areas for very short periods, until they attained 6 months of age. Routinely the flocks were vaccinated against Peste-des-petitis ruminants (PPR), Foot and mouth (FMD), and enterotoxaemia (ET). Targeted deworming were carried out during the pre-monsoon season (May to June) and in the post-monsoon season (September to October) for the control of gastrointestinal nematodes. The study area had semi-arid climate and average annual rainfall was about 375 mm, spread across the months of June to September. The soil was sandy with natural pasture and bush as the main vegetation type. The pastures were mainly *Cenchrus ciliaris* and *C. setigerus*, along with native annual and perennial flora. Meteorological data indicated that the temperature varied from 4.0 °C to 24.3 °C during winter and 27.5 °C to 42.4 °C during summer.

The phenotypic data comprised of 2217 records from does milked twice daily. The traits analysed were estimated milk yield at 90 days (MY 90), milk yield at 140 days (MY 140), total milk yield per lactation (TMY) and lactation length (LL). Milk yield was recorded every seven days until the animals became dry. The estimated milk yield for different periods were calculated using Fleischmann’s method (Ruiz et al., 2000) with some modification as follows:

TMY = P_1_ × D_1_ + (k_i=2_((P_i_ + P_i+1_)/2) × D_i_) + P_k+1_ × 3.5

Where D_1_ represents the interval between birth/kidding and first recording, D_i_ represents any subsequent interval between two recordings, P_i_ represents the yields of record i and the record (i + 1) with i = (1,…., k), and 3.5 assumed be the number of days between the last recording and drying off.

Pedigree information was available for 1217 animals, which were the progeny of 173 sires and 466 dams bred across 7 generations.

### 2.2 Statistical analysis

Initially data were explored for summary statistics and normality using SAS (SAS, 2013). We fitted a univariate normal plot function that uses both numerical and graphical methods to test for normality. The Shapiro-Wilk test (Shapiro and Wilk, 1965) for sample size less than 2000 was used test for normality (SAS, 2013).

The estimates of (co)-variance components were obtained using the *ASReml* programme (Gilmour et al., 2009) fitting mixed linear models accounting for environmental effects of parity (1–6+), year (1990–2013), season (autumn and spring) and type of birth (single and multiple) as fixed effects and effects of animal, maternal and permanent environmental effects due to the animal fitted as random effects. Initially univariate models were fitted:

(1) ***Model 1: y*** ***=*** ***Xb*** ***+*** ***Z_a_*** ***+*** ***Zpe*** ***+*** ***e***

(2) ***Model 2: y*** ***=*** ***Xb*** ***+*** ***Z_a_*** ***+*** ***Z_m_*** ***+*** ***Zpe*** ***+*** ***e***

where *y* is a vector of observations on specific traits of the animal; ***b*** is a vector of fixed effects; ***a, m*** *and **pe*** are vectors of random effects describing additive genetic effects, maternal additive effects and permanent environment effects due to animal; **X**, **Z** are corresponding incidence matrices relating to each effect to **Y**; and ***e*** is the vector of residuals. To compare different random effects, log likelihood ratio tests (LRT) were carried out to determine the most suitable model for each trait in univariate analyses (Morrell, 1998).The test statistic was −2[lnL (2)-lnL (1)] where L (n) is the log likelihood of Model n. Critical values for the LRT were taken from a mixture distribution ½ (1) and ½ (0) (Self and Liang, 1987). The relationship matrix was constructed using pedigree records. The narrow-sense heritabilities (h^2^) for example were estimated as follows: animal model: *h^2^* *=* σ*^2^_additive_/*(σ*^2^_additive_* *+* σ*^2^_pe_* *+* σ*^2^_maternal_* *+* σ*^2^_residual_*) and repeatabilities (r) were calculated as r = (σ*^2^_additive_* *+* σ*^2^_pe_*)/(σ*^2^_additive_* *+* σ*^2^_pe_ +σ^2^_maternal_* *+* σ*^2^_residual_*).

The genetic trends were estimated for MY90, MY 140 and TMY obtained by regressing the means of estimated breeding values (ebv) on year of birth weighted by the number of animals in each year. Similarly, the phenotypic trends were estimated by regressing least squares means of milk production traits on year of birth. These procedures were carried out using the SAS computer package (SAS, 2013).

## 3 Results and discussion

The summary statistics for milk yield traits are presented in Table 1. The means for MY90, MY 140 and TMY were 80.2, 114.0 and 124.8 kg, respectively for Jamunapari goat. The average lactation length was 179.5 days, which was longer than other Indian breeds. Jamunapari goats were productive until seventh parity; however, some goats were still productive until 11th parity. It has been observed that the influence of the season of kidding on milk yield at MY90, MY140 and TMY was significant (P < 0.01). Parity had significant effect (P < 0.01) on milk yield over the years. The year of birth and kidding year had significant effects (P < 0.01) on milk yield traits. Does with multiple births produced more milk compared to those bearing singles.

**Table 1:** The summary statistics (means, standard deviations and standard errors) for milk yield traits (kg) and lactation length (in days) in Jamunapari goats.

	MY 90 days	MY140 days	Total Milk yield	Lactation Length
Number of Records	2217	1788	2099	2099
Number of years	24	24	24	24
				
Mean	80.18	113.98	124.82	179.50
SD	33.3	38.1	51.06	42.17
Standard error	0.71	0.90	1.11	0.92
CV (%)	41.6	33.48	40.90	23.49
Range	21.8–168.0	46.8–233.6	33.0−273.7	70–277

The animal model (Model 1) fitting the permanent environment due to the animal was the most appropriate model for milk yield at 90 and 140, and LL with model 2 only most appropriate for TMY (Table 2). The parameter estimates for milk yield and lactation length are presented in Table 2. The estimates of direct additive heritability for MY90, MY140 and TMY were low to moderate and ranged from 0.15 to 0.28 (Table 2). The maternal variance contributed significantly for TMY and was low for MY90 and MY 140. The permanent environmental component due to animal and litter contributed negligibly. The heritability estimates across different traits were significantly (P < 0.05) different from zero with small standard errors (varies from 0.02 to 0.08). This is mainly because of large sample size and indicating that the genetic improvement by selection for milk production for 90 days and 140 days is likely to be successful.

**Table 2:** Model effect and genetic parameters of milk yield traits in Jamunapari goats.

	MY90	MY140	LMY	LL
Models	1	1	2	1
Parameter				
σ^2^_a_	5703.29	27002.8	35325.8	33.4
σ^2^_pe_	20250.4	43445.9	61547.6	245.9
σ^2^_maternal_	–	–	18404.6	–
σ^2^_residual_	12155.5	26077.3	24724.5	1128.4
σ^2^_phenotypic_	38109	96526.0	140000.0	1407.6
se	1613.1	4950.0	7550.7	46.1
h^2^_additive_	0.15	0.28	0.25	0.02
se	0.05	0.07	0.08	0.03
repeatability	0.68	0.73	0.69	0.20
se	0.02	0.02	0.05	0.03
mat^2^			0.13	
se			0.05	
Log L	−2182.50	−10561.6	−2492.28	−8544.21

Where σ^2^_a_ ∼ direct additive variance, σ^2^_maternal_ ∼ maternal variance, σ^2^_pe_ ∼ variance of permanent environment due to the animal, σ^2^_residual_ ∼ residual variance, σ^2^_phenotypic_ ∼ total phenotypic variance, −2logL ∼ Log likelihood ratio test; s.e. ∼ standard error; h^2^_additive_ ∼ direct additive heritability; h^2^_maternal_ ∼ maternal heritability; repeatability ∼ permanent environment due to animal variance. Model 1 ∼ fitting additive and permanent environment due to the animal, Model 2 ∼ fitting additive, maternal effects and permanent environment due to the animal

The heritability estimates from the present study were comparable with the following reports in literature (Boichard et al., 1989, Belichon et al., 1999, Muller et al., 2002, Weppert and Hayes, 2004, Rupp et al., 2011). Boichard et al., 1989 reported heritability estimates of 0.29–0.39 for milk yield in French Alpine and Saanen primiparous goats and Belichon et al. (1999) reported heritability estimates for milk yield in Alpine goats from 0.34 to 0.37 and in Saanen from 0.32 to 0.40 in France. Muller et al. (2002) reported heritability estimates of 0.23 for milk yield of South African Saanens. Weppert and Hayes (2004) estimated heritability estimates of 0.19 for milk yield in Alpine, Toggenburg, Saanen and Nubian breed. Montaldo et al. (2010a) estimated heritability for first-parity milk yield of US dairy goats as 0.36 across breeds, which varied within breed from 0.35 to 0.38. Similarly, low to moderate estimates of heritability (0.17–0.30) for milk yield in Saanen goats were reported in central Mexico (Valencia et al., 2007, Torres-Vázquez et al., 2009). Rupp et al. (2011) also reported heritability of 0.30 for US Alpines and Saanens for first lactation yield. Low heritability estimates of 0.04 for lactation length was reported by Montaldo et al. (2010a) in Saanen goats reared in Mexico. We also obtained low estimates of heritability for LL. The heritability estimates from our study were lower to those reported in other goat breeds, 0.68, 0.61, and 0.54 for Alpines, Saanens and Toggenburgs, respectively (Kennedy et al., 1982).

Repeatability estimates for all milk production traits were high ranging from 0.68, 0.73 and 0.69 for MY90, MY140 and TMY, respectively. The repeatability of milk production traits was higher than those reported in literature (Ilahi et al., 1998, Morris et al., 2006). Ilahi et al. (1998) estimated the heritability of 0.32 and a repeatability of 0.53 for milk yield of French Alpines. Morris et al. (2006) reported heritability estimates of 0.35 (repeatability of 0.52) for daily milk yield in New Zealand Saanens goats.

The additive maternal genetic effects were low and only important for TMY. This may be due to environmental influence of dams to their kids from conception to birth via intrauterine environment and from birth to 3 months of age via maternal colostrum feeding and milk suckling. Maternal genetic effects have been described in domestic mammals such as swine (Southwood and Kennedy, 1990) and beef cattle (Dodenhoff et al., 1999). However, other studies have reported that maternal genetic effects were not important in milk yield traits for dairy cattle (Khattab et al., 2005, Albuquerque et al., 1995, Schutz et al., 1992).

**Fig. 1:**
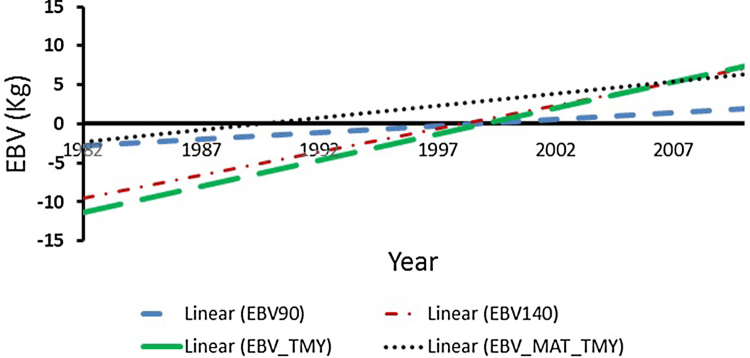
Direct and maternal genetic trends of milk yield traits in Jamunapari goats in semi-arid tropic (black dotted line for MY90 (EBV90), red dashed and dotted line for MY140 (EBV140), green dashed line for TMY (EBV_TMY) and a black dotted line maternal genetic trend of total milk yield (EBV_MAT_TMY) from 1982 to 2013 period). (For interpretation of the references to colour in this figure legend, the reader is referred to the web version of this article.)

A positive genetic trend was observed for milk yield at 90 days, 140 days and TMY in Jamunapari goat population. Genetic trends for milk yield traits MY90, MY140 and TMY are presented in Fig. 1. There was increase in mean milk yield of 0.25, 0.70 and 0.72 kg/year at 90 days, 140 days and TMY, respectively in Jamunapari goat. The maximum limit of increase in milk yield was 0.70, 1.63 and 1.99 kg per year in MY90, MY140 and TMY, respectively. The maternal genetic trend was positive and was 0.42 kg/year for TMY (Fig. 1). The total genetic progress was estimated as total change in mean estimated breeding values in 2013 from those estimated in 1990 expressed as proportion of genetic standard deviation. The total genetic gain as proportion of genetic standard deviation (σ_A_) for TMY was 1.76. Genetic trend of milk production in Alpine and Saannen goats was 13.6 L/year and 12.5 L/year in France during 1990–2000. Similarly, the genetic trends in Alpine and Saannen goat were 8.6 L/year and 7.0 L/year in USA during 1995–2000 (Clement et al., 2002). Also, a positive genetic trend of 7.0 kg per year was obtained for 305 days mature equivalent milk yield in the female Saanen goat population in the US between 1995 and 2000 (Wiggans et al., 2003). This trend is equivalent to an annual genetic progress of 0.79% in average milk production (Montaldo and Manfredi, 2002). Similarly, the phenotypic trend was positive for all the milk production traits (Supplementary material Fig. 1). The average increase in phenotypic trend for MY90, MY140 and TMY was 1.2, 4.4 and 5.4 kg/year, respectively. Genetic trends and phenotypic trends for MY90, MY140 and TMY were positive and indicated significant improvement in lactational milk production performance due to selective breeding. Results of this study suggest that the positive genetic trends for milk production were due to an effective but low selection response in Jamunapari goats using natural mating in this flock. Moreover, there is still more room for management during pregnancy period to improve milk production and improving intra-uterine environment for increasing the genetic merit of the animals.

## 4 Conclusions

Estimates of repeatability and heritability were moderate to high indicating that the successful selection is feasible for improving milk yield traits. Genetic trends and phenotypic trends for MY90, MY140 and TMY were positive and indicated significant improvement in lactational milk production performance due to selective breeding.

## Conflict of interest

The authors declared no conflict of interest

## Figures and Tables

**Figure:**
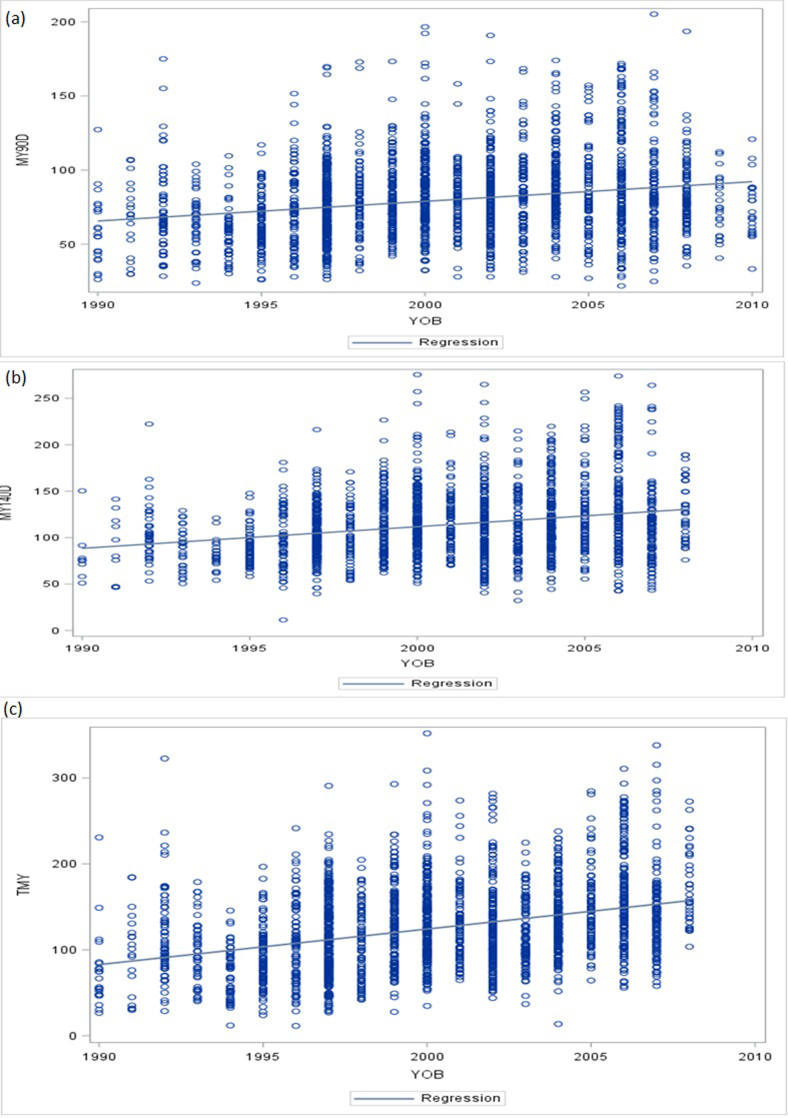


## References

[bib0005] Albuquerque L.G., Dimov G., Keown J.F. (1995). Estimates using an Animal Model of (Co) variances for yields of milk, fat and protein for the first lactation of Holstein cows in California and New York. J. Dairy Sci..

[bib0010] Andonov S., Odegard J., Boman I.A., Svendsen M., Holme I.J., Adnoy T., Vukovic V., Klemetsdal G. (2007). Validation of test-day models for genetic evaluation of dairy goats in Norway. J. Dairy Sci..

[bib0015] Barillet F. (2007). Genetic improvement for dairy production in sheep and goats. Small Rumin. Res..

[bib0020] Belichon S., Manfredi E., Piacere A. (1999). Genetic parameters of dairy traits in the Alpine and Saanen goat breeds. Genet. Sel. Evol..

[bib0025] Boichard D., Bouloc N., Ricordeau G., Piacere A., Barillet F. (1989). Genetic parameters for first lactation dairy traits in the Alpine and Saanen goat breeds. Genet. Sel. Evol..

[bib0030] Brito L.F., Silva F.G., Melo A.L.P., Caetano G.C., Torres R.A., Rodrigues M.T., Menezes G.R.O. (2011). Genetic and environmental factors that influence production and quality of milk of Alpine and Saanen goats. Genet. Mol Res..

[bib0035] Clement V., Boichard D., Piacere A., Barbat A., Manfredi E. (2002). Genetic evaluation of French goats for dairy and type traits. Proc. 7th World Congress Applied in Livestock Production, Montpellier, 19–23 August 2002.

[bib0040] Devendra C., Liang J.B. (2012). Conference summary of dairy goats in Asia: status, multifunctional contribution to food security and potential improvements. Small Rumin. Res..

[bib0045] Devendra C. (2012). Multifunctional relevance and contribution of dairy goats to food and nutrition security. Proc. First Asian Dairy Goat Conf..

[bib0050] Dodenhoff J., Vamvleck L.D., Gregory K.E. (1999). Estimation of direct: maternal and grand maternal genetic effects for weaning weight in several breeds of beef cattle. J. Anim. Sci.

[bib0055] Gilmour A.R., Cullis B.R., Welham S.J., Thompson R. (2009). ASREML Discovery Reference Manual.

[bib0060] Haenlein G.F.W. (2004). Goat milk in human nutrition. Small Rumin. Res..

[bib0065] Ilahi H., Chastin P., Martin J., Monod F., Manfredi E. (1998). Genetic association between milking speed and milk production.

[bib0070] Kala S.N., Prakash B. (1990). Genetic and phenotypic parameters of milk yield and milk composition in two Indian goat breeds. Small Rumin. Res..

[bib0075] Kennedy B.W., Finley C.M., Bradford G.E. (1982). Phenotypic and genetic relationships between reproduction and milk production in dairy goats. J. Dairy Sci..

[bib0080] Khattab A.S., Atil H., Badawy L. (2005). Variances of direct and maternal genetic effects for milk yield and age at first calving in a herd of Friesian cattle in Egypt. Arch. Tierz. Dummerstorf..

[bib0085] Majid A.M., Cartwright T.C., Yazman J.A., FitzhughJr H.A. (1994). Performance of five breeds of dairy goats in southern United States. II. Lactation yield and curves. World Rev. Anim. Prod..

[bib0090] Mavrogenis A.P., Papachristoforou C., Lysandrides P., Roushias A. (1989). Environmental and genetic effects of udder characteristics and milk production in Damascus goats. Small Rumin. Res..

[bib0095] Montaldo H.H., Manfredi E. (2002). Organisation of Selection Programmes for Dairy Goats. Commun. No. 01–35. Proc. 7th World Congr. Genet. Appl. Livest. Prod, Montpellier, France. Institut National de la RechercheAgronomique (INRA).

[bib0100] Montaldo H.H., Valencia-Posadas M., Wiggans G.R., Shepard L., Torres-Vazquez J.A. (2010). Short communication: genetic and environmental relationships between milk yield and kidding interval in dairy goats. J. Dairy Sci..

[bib0105] Montaldo H.H., Torres-Hernandez G., Valencia-Posadas M. (2010). Goat breeding research in Mexico. Small Rumin. Res..

[bib0110] Morris C.A., Wheeler M., Lanuzel M. (2006). Genetic trend and parameter estimates for milk yield traits and kidding date in a Saanen goat herd in New Zealand. J. Agr. Res..

[bib0115] Muller C.J.C., Cloet S.W.P., Schoeman S.J. (2002). Estimation of genetic parameters for milk yield and milk composition of South African Saanen goats. Commun.No. 01-52. Proc. 7th World Congr.Genet.Appl. Livest.Prod., Montpellier, France.I Nstitut National De La Recherche Agronomique (INRA).

[bib0120] Rabasco A., Serradilla J.M., Padilla J.A., Serrano A. (1993). Genetic and non-genetic sources of variation in yield and composition of milk in Verata goats. Small Rumin. Res..

[bib0125] Rout P.K., Saxena V.K., Khan B.U., Roy R., Mandal A., Singh S.K., Singh L.B. (2000). Characterization of Jamunapari goats in their home tract. Anim. Genet. Res. Inf..

[bib0130] Rout P.K., Singh M.K., Roy R., Sharma N., Haenlein G.F.W. (2004). Jamunapari-a dairy goat breed in India. Dairy Goat J. (USA)..

[bib0135] Rupp R., Clément V., Piacere A., Robert-Granie C., Manfredi E. (2011). Genetic parameters for milk somatic cell score and relationship with production and udder type traits in dairy Alpine and Saanen primiparous goats. J. Dairy Sci..

[bib0140] SAS (2013). Online Doc 9.1.3.

[bib0145] Schutz M.M., Freeman A.E., Beitz D.C., Mayfield J.E. (1992). The importance of maternal lineage on milk yield traits of dairy cattle. J. Dairy Sci..

[bib0150] Selvaggi M., Dario C. (2015). Genetic analysis of milk production traits in Jonica goats. Small rumin. Res..

[bib0155] Serradilla J.M. (2001). Use of high yielding goat breeds for milk production. LivestProd Sci..

[bib0160] Shapiro S.S., Wilk M.B. (1965). An analysis of variance test for normality (complete samples). Biometrika.

[bib0165] Southwood O.I., Kennedy B.W. (1990). Estimates of direct and maternal genetic variance for litter size in Canadian Yorkshire and Landrace Swine using an Animal Model. J. Anim. Sci.

[bib0170] Sullivan B.P., Kennedy B.W., Schaeffer L.R. (1986). Heritabilities, repeatabilities and correlations for milk, fat and protein yields in goats. J. Dairy Sci..

[bib0175] Torres-Vázquez J.A., Valencia-Posadas M., Castillo-Juárez H., Montaldo H.H. (2009). Genetic and phenotypic parameters of milk yield: milk composition and age at first kidding in Saanen goats from Mexico. Livest. Sci..

[bib0180] Torres-Vazquez J.A., Valencia-Posadas M., Castillo-Juarez H., Montaldo H.H. (2010). Genetic and phenotypic trends for milk yield and milk composition traits of Saanen goats from Mexico. Revista Mexicana de CienciasPecurias..

[bib0185] Valencia M., Dobler J., Montaldo H.H. (2007). Genetic and phenotypic parameters for lactation traits in a flock of Saanen goats in Mexico. Small Rumin. Res..

[bib0190] Weppert M., Hayes J.F. (2004). Direct genetic and maternal genetic influences on first lactation production in four breeds of dairy goats. Small Rumin. Res..

[bib0195] Wiggans G.R., Van Dijk J.W., Misztal I. (1988). Genetic evaluation of dairy goats for milk and fat yield with an animal model. J. Dairy Sci..

[bib0200] Wiggans G., Hubbard S.M., Wrigth J.R. (2003). AIPL Goat Evaluation Description. Genetic Evaluation of Dairy Goats for Yield and Type. http://www.aipl.arsusda.gov/reference/goat/goatsfs.html.

[bib0205] Wiggans G.R. (1989). Animal model evaluation of dairy goats for milk, fat, and protein yields with crossbred animals included. J. Dairy Sci..

