# Supplementary material for: Estimation of genetic parameters and genetic trends for milk yield traits in Jamunapari goats in semiarid tropics
Source: Small Rumin Res. 2017 Aug;153:62–5. doi: 10.1016/j.smallrumres.2017.05.004 (PMC5555442; doi:10.1016/j.smallrumres.2017.05.004)
Supplement: Supplementary file 1 [file mmc1.doc]

**Supplementary material Figure 1 (a, b, c).** Phenotypic trend of MY90, MY 140 and TMY in Jamunapari goats in semi-arid tropic .
